# The effect of nebulized salbutamol or isotonic saline on exercise-induced bronchoconstriction in elite skaters following a 1,500-meter race: study protocol for a randomized controlled trial

**DOI:** 10.1186/1745-6215-14-204

**Published:** 2013-07-09

**Authors:** Jean MM Driessen, Margryt Gerritsma, Jaap Westbroek, Nick HT ten Hacken, Frans HC de Jongh

**Affiliations:** 1 Sports Medicine, Tjongerschans Hospital, Heerenveen, the Netherlands; 2 Sport and Management, Hanzehogeschool, Groningen, the Netherlands; 3 Pulmonology, Tjongerschans Hospital, Heerenveen, the Netherlands; 4 Pulmonology, University Medical Centre Groningen, Groningen, the Netherlands; 5 Pulmonology, Medisch Spectum Twente, Enschede, the Netherlands; 6 Pediatric Pulmonology, Academic Medical Centre, Amsterdam, the Netherlands

**Keywords:** Elite athletes, Exercise-induced bronchoconstriction, Salbutamol, Forced oscillation technique, Small airways

## Abstract

**Background:**

Prevalence of exercise-induced bronchoconstriction (EIB) is high in elite athletes, especially after many years training in cold and dry air conditions. The primary treatment of EIB is inhaling a short-acting beta-2-agonist such as salbutamol. However, professional speed skaters also inhale nebulized isotonic saline or tap water before and after a race or intense training. The use of nebulized isotonic saline or tap water to prevent EIB has not been studied before, raising questions about safety and efficacy. The aim of this study is to analyze the acute effect of nebulized isotonic saline or salbutamol on EIB in elite speed skaters following a1,500-meter race.

**Methods:**

This randomized controlled trial compares single dose treatment of 1 mg nebulized salbutamol in 4 mL of isotonic saline, or with 5 mL of isotonic saline. A minimum of 13 participants will be allocated in each treatment group. Participants should be between 18 and 35 years of age and able to skate 1,500 m in less than 2 min 10 s (women) or 2 min 05 s (men). Repeated measurements of spirometry, forced oscillation technique, and electromyography will be performed before and after an official 1,500-m race. Primary outcome of the study is the difference in fall in FEV_1_ after exercise in the different treatment groups. The trial is currently enrolling participants.

**Discussion:**

Elite athletes run the risk of pulmonary inflammation and remodeling as a consequence of their frequent exercise, and thus increased ventilation in cold and dry environments. Although inhalation of nebulized isotonic saline is commonplace, no study has ever investigated the safety or efficacy of this treatment.

**Trial registration:**

This trial protocol was registered with the Dutch trial registration for clinical trials under number NTR3550

## Background

Exercise-induced bronchoconstriction (EIB) is the most common chronic condition in elite athletes [1]. Especially endurance athletes who reach high ventilation in cold, dry air on a regular basis are susceptible to EIB [1,2]. A study using a canine model highlighted the negative effect of dry air on the airways as it leads to airway inflammation and airway remodeling [3]. In humans this find has been confirmed in bronchial biopsies from competitive elite skiers, showing signs of eosinophilic and neutrophilic airway inflammation in the lungs, similar to those seen in asthma [4].

In elite speed skaters intense training may cause thoracic pain and cause EIB [5]. For this reason many speed skaters use a roll-collar in training to warm and humidify inhaled air. Beuther *et al.* already showed that warming the inhaled air can reduce EIB [6]. In competition, due to aerodynamics, this method cannot be used. In an effort to reduce EIB and thoracic pain, international professional speed skaters nowadays inhale nebulized isotonic saline or tap water before and after a race or intense training session. In EIB, the primary treatment is a short-acting beta-2-agonist such as salbutamol [1,7]. This treatment reduced smooth muscle contraction very effectively, but still is an example of symptomatic therapy. The recommended dosage of nebulized salbutamol is 5 mg, which would require a therapeutic use exemption for participating athletes from the world anti-doping agency (WADA) (http://www.wada-ama.org). Although such a dosage is safe in highly trained athletes [8], we routinely recommend using 1 mg of salbutamol, which we believe leads to substantial reductions in EIB in this population. The use of nebulized isotonic saline treat EIB has not been studied before, raising questions about safety and efficacy.

Spirometry before and after exercise is the golden standard for assessing the occurrence and severity of EIB [7]. A fall in forced expiratory volume in 1 s (FEV_1_) >10%, after exercise (>95% predicted heart frequency) indicates EIB. A potential disadvantage of spirometry is the use of a forced maneuver and not everybody is able to perform reliable measurements. In addition the maneuver requires a forced deep inspiration which in asthmatic children with EIB may lead to bronchodilation [9], and an underestimation of EIB. The forced oscillation technique (FOT) may be used to evaluate the patency of the airways without the need of forced breathing [10]. Another substitute for evaluating lung function without the need of forced breathing may be electromyography (EMG), however, it has not been used to evaluate EIB [11,12].

The aim of the study is to analyze the effect of nebulized isotonic saline or salbutamol on EIB in elite speed skaters following a 1,500-m race using spirometry, FOT, and EMG.

## Methods

### Design

**Figure 1:**
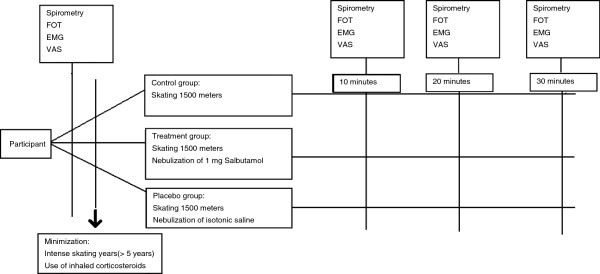
**Timing if randomization and data collection of the study.** EMG, Electromyography; FOT, Forced oscillation technique; VAS, Visual analogue scale.

This study is a prospective, randomized, double-blind, placebo, treatment, and non-intervention controlled study, consisting of three parallel groups. Participants will be randomized to receive either no intervention (control), 1 mg of nebulized salbutamol (treatment), or nebulized isotonic saline (placebo). An overview of the timing of randomization and data collection can be seen in Figure 1.

This trial protocol was ethically approved by the Regional Ethical Committee (RTPO), Leeuwarden (primary) and the Central Committee on Research Involving Human Subjects (CCMO), The Hague (secondary) and was registered with the Dutch trial registration for clinical trials (http://www.trialregister.nl) under number NTR 3550. All participants signed a written informed consent form.

### Setting and participants

Participants are recruited from the provincial selection teams and professional skating teams in the Netherlands. All skaters in these teams will be invited to participate in the study after they will be screened for eligibility.

### Inclusion criteria

Skaters should be between 18 and 35 years of age and be able to skate the 1,500 m in less than 2 min 10 s (women) and 2 min 05 s (men), and be able to perform lung function tests according to current standards [13].

### Exclusion criteria

Skaters will be excluded if they had: a respiratory infection within 6 weeks prior to the tests for which medication has been prescribed; an FEV_1_ <70% of predicted; ultrasonic nebulization with tap water or saline solutions within 48 h before testing; use of short-acting beta agonists 8 h before exercise; use of long-acting beta agonists 24 h before exercise; use of a leukotriene-receptor-antagonist 36 h before exercise.

### Interventions

**Figure 2:**
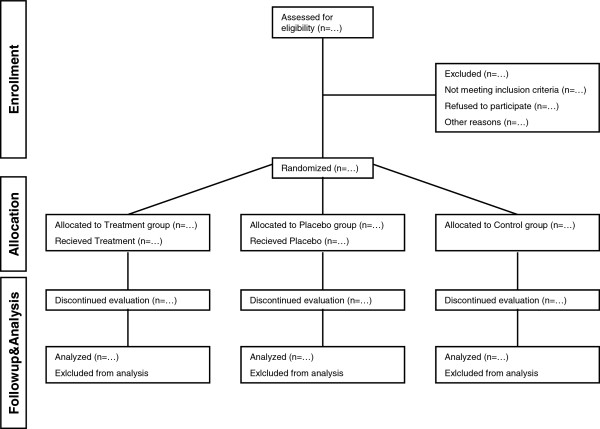
A flow diagram of the progress through the phases of the study.

Four minutes after completing the 1,500-m race, the elite skater will either inhale nebulized salbutamol (1 mg), nebulized isotonic saline for 5 min or they will not receive an intervention. An elite athlete inhaling nebulized tap water can be seen in Figure 2. The dose of salbutamol, 1 mg, will be used to stay well below the WADA cutoff for doping at an elite level of 1.6 mg daily. The route of administration has been chosen to allow a viable comparison with nebulized saline [14]. Inhaled Salbutamol has a longstanding record for treatment of bronchoconstriction and poses a minimal risk for cardiovascular complications even if used in dosages above the recommended dosage [15]. The dosage used is below the recommended dosage, furthermore, expert opinion states that salbutamol has been used in higher dosages immediately after extremely strenuous activity before and no adverse reactions have been reported. Furthermore, in a study by Elers *et al*., high dosages of inhaled salbutamol were used to evaluate anabolic effects in well trained athletes (VO_2_ max 66 mg∙ml∙min^-1^). No beneficial or harmful effects were seen on cardiopulmonary function [8].

### Pulmonary function measurements

FOT measurements will be performed with R.O.S., Oscilink®, Sensormedics® to measure general respiratory resistance and reactance. FOT measurements will consist of three repeated measurements with nose clipped and with hands supporting cheeks and base of the mouth [10]. FOT measurements will be performed before and at 10, 15, and 30 min after exercise. The average of resistance and reactance values will be used for statistical analysis.

A Masterscope® Jaeger®, will be used to measure flow-volume loops in accordance with current ERS/ATS guidelines [13]. Lung function will be calculated from the best curve. Flow volumes will be measured in duplicate before and at 11, 16, and 31 min after exercise, the best values at each time point retained for analysis. Feeling of dyspnea and thoracic pain will be evaluated using a visual analogue scale (VAS) after every complete spirometry measurement.

EMG of the diaphragm and intercostal muscles will be derived transcutaneously from pairs of single electrodes (disposable Neotrode, ConMed Corporation, NY) [11]. To obtain the electrical activity of the diaphragm, two electrodes will be placed bilaterally below the costal margin in the nipple line (frontal lead of diaphragm) and two bilaterally on the back at the same level (dorsal lead of diaphragm). The mean value of the processed data of the frontal and dorsal leads of the diaphragm represented the electrical activity of the whole diaphragm. A common electrode will be placed at the height of the sternum.

### Exercise provocation challenge

Exercise testing for measuring EIB will be performed by skating 1,500 m. Cold, dry air will be obtained while testing in the local skating ring, IJsbaan Thialf, Heerenveen (http://www.thialf.nl).

### Outcomes

The primary outcome of the study is the difference in percentage fall in FEV_1_ from baseline after exercise in the different treatment groups. Secondary outcomes of the study are the difference in airway resistance and reactance in kPa/l/s at low frequency as measured with the FOT and difference in diaphragm and intercostal muscle activity in the logarithm of mean bottom ratio of respiratory muscle activity between the different treatment groups.

### Sample size

Numbers of study participants were calculated using the calculations of Dupont, and setting power of the study at 80% and *P*=0.05 [16]. Data used to produce these numbers came from research performed by Driessen *et al.*[17]. We expect no difference between placebo and control groups. Analyzing the protective effect of salbutamol in a randomized control trial, with an expected difference in FEV_1_ of 10% and a standard deviation from the mean of 6% the number of participants was set at 13 per group. Therefore the total number of randomized participants will be at least 39. In the aforementioned research performed by Driessen *et al.*, 95% of patients completed the study. Therefore the enrolled patient count will be 1.05 times the total number (39•1.05=41).

### Randomization

Participants are randomized using a pre-generated randomization list. For randomization a linear congruential algorithm of Park and Miller with Bays-Durham shuffling was employed, using block sizes of two, four, and eight participants. Stratified minimization will commence using the number of intensive training years (> or <5 years) and the current use of asthma medication. A flow diagram of the progress through the phases of the study can be seen in Figure 1.

### Blinding

The study medication will be delivered to the skating ring in a standard syringe, containing either 1 mg salbutamol in isotonic saline or solely isotonic saline with a standard label. The lack of inhalation in the control group cannot be masked. Assessment of lung function will be assessed without knowledge of the received treatment.

### Discontinuation

Participants can leave the study at any time for any reason if they wish to do so without any consequences. The investigator can decide to withdraw a participant from the study for urgent medical reasons (for example, a fall in FEV_1_ >25% from baseline). Discontinued participants will not be replaced.

### Data analysis

Best values of spirometric measurements, mean values of FOT measurements and EMG measurements recordings of 60 s during FOT measurements will be used for statistical calculations. Data will consist of a set of comparing participants who nebulized 1 mg Salbutamol, isotonic saline, and one set of controls. Once gathered, data will be analyzed with SPSS analytical software after testing for normality with a Shapiro-Wilk test. Difference between groups will be analyzed with a chi-square test for dichotomous variables and a one-way ANOVA, followed by a post-hoc Tukey’s test for continuous variables.

### Data storage

Data will be encoded in Access® for windows. Traceable participant data will be coded in a separate file, data used in analysis will not be traceable to the participant without the coding file. Data will be entered using the double data entry format.

## Discussion

This trial investigates a common procedure in elite athletes. Elite athletes run the risk of pulmonary inflammation and remodeling as a consequence of their frequent exercise, and thus increased ventilation in cold and dry environments [3,4,7]. Although inhalation of nebulized isotonic saline is commonplace, no study has ever investigated the safety or efficacy of this treatment.

Furthermore the pulmonary response to such an extreme performance has not been investigated in such detail, using not only spirometry and feeling of dyspnea, but also the FOT and EMG of breathing musculature.

There are some limitations to this study. First and foremost is the inclusion of the participants limited to elite skaters. Not all speed skaters however will show a substantial drop in FEV_1_ after exercise limiting the power of our study. In our experience however, at least 40% of elite speed skaters in the Netherlands will show a substantial drop in FEV_1_ after exercise, this is even higher than the percentage of American elite winter athletes experiencing EIB [2]. Furthermore the inclusion of the FOT and EMG measurements will allow more subtle evaluation of pulmonary function, not disrupted by forced breathing [9-11]. We will be able to analyze the smaller airways using the extensive pulmonary function measurements, however we preferred to also assess the damage to pulmonary parenchyma using Clara Cell protein (CC16) [18]. We believe however that analyzing CC16 in serum would have severely hampered the inclusion rate of the participants.

## Trial status

The trial is currently enrolling participants and collects data on three separate dates; all test dates will be official races, approved by the Royal Dutch Skating organization (http://www.knsb.nl). The first test date will be 21 December 2012. The second test date is planned in February 2013. A third test date will be added in the fall of 2013. Data analysis is expected to be completed in December 2013.

## Abbreviations

CCMO: Centrale Commissie voor Mensgebonden Onderzoek; EIB: Exercise-induced bronchoconstriction; EMG: Electromyography; FOT: Forced oscillation technique; RTPO: Regionaal Toetsingsorgaan voor Persoonsgebonden Onderzoek; VAS: Visual analogue scale; WADA: World anti-doping agency

## Competing interests

This clinical trial was founded by a grant by the pulmonology departments of the University Medical Centre Groningen, Medical Spectrum Twente and Tjongerschans Hospital. The authors declare to have no competing interests.

## Authors’ contributions

JD, MG, JW, NtH, and FdJ all made substantial contributions on the design of the trial. JD wrote the draft manuscript with substantial input of MG. All authors provided critical review of the manuscript and approved the final version.

## Authors’ information

JD is a sports physician in training with a special interest in EIB. MG is a professional speed skater and is graduating as a BSc in Sport and Management in July 2013 (Hanzehogeschool Groningen). JW is a chest physician with a special interest in sports medicine and EIB; he has been the team physician for several professional speed skating teams. NtH is a chest physician with special interest in airway inflammation in nocturnal asthma and EIB. FdJ is a physiologist with expert knowledge on lung function measurements, pulmonary physiology, medical aerosols, and pulmonary distribution of nebulized medication.

## References

[B1] Carlsen KH Anderson SD Bjermer L Bonini S Brusasco V Canonica W Cummiskey J Delgado L Del Giacco SR Drobnic F Haahtela T Larsson K Palange P Popov T van Cauwenberge P European Respiratory Society; European Academy of Allergy and Clinical Immunology Exercise-induced asthma, respiratory and allergic disorders in elite athletes: epidemiology, mechanisms and diagnosis: part I of the report from the Joint Task Force of the European Respiratory Society (ERS) and the European Academy of Allergy and Clinical Immunology (EAACI) in cooperation with GA2LEN Allergy 2008 63 387 403 10.1111/j.1398-9995.2008.01662.x 18315727

[B2] Wilber RL Rundell KW Szmedra L Jenkinson DM Im J Drake SD Incidence of exercise-induced bronchospasm in Olympic winter sport athletes Med Sci Sports Exerc 2000 32 732 737 10.1097/00005768-200004000-00003 10776890

[B3] Davis M Mckiernan B McCullough S Nelson S Mandsager R Willard M Dorsey K Racing Alaskan sled dogs as a model of “ski asthma” Am J Respir Crit Care Med 2002 15 878 882 12231501 10.1164/rccm.200112-142BC

[B4] Karjalainen E Laitinen A Sue-Chu M Altraja A Bjermer L Laitinen L Evidence of airway inflammation and remodeling in ski athletes with and without bronchial hyperresponsiveness to methacholine Am J Respir Crit Care Med 2000 161 2086 2091 10.1164/ajrccm.161.6.9907025 10852791

[B5] Paul DW Bogaard JM Hop WC The bronchoconstrictor effect of strenuous exercise at low temperatures in normal athletes Int J Sports Med 1993 14 433 436 10.1055/s-2007-1021205 8300267

[B6] Beuther DA Martin RJ Efficacy of a heat exchanger mask in cold exercise-induced asthma Chest 2006 129 1188 1193 10.1378/chest.129.5.1188 16685008

[B7] Anderson SD Kippelen P Assessment and prevention of exercise-induced bronchoconstriction Br J Sports Med 2012 46 391 396 10.1136/bjsports-2011-090810 22247297

[B8] Elers J Mørkeberg J Jansen T Belhage B Backer V High-dose inhaled salbutamol has no acute effects on aerobic capacity or oxygen uptake kinetics in healthy trained men Scand J Med Sci Sports 2012 22 232 239 10.1111/j.1600-0838.2010.01251.x 21083771

[B9] Schweitzer C Vu LT Nguyen YT Choné C Demoulin B Marchal F Estimation of the bronchodilatory effect of deep inhalation after a free run in children Eur Respir J 2006 28 89 95 10.1183/09031936.06.00114705 16571612

[B10] Oostveen E MacLeod D Lorino H Farré R Hantos Z Desager K Marchal F ERS Task Force on Respiratory Impedance Measurements The forced oscillation technique in clinical practice: methodology, recommendations and future developments Eur Respir J 2003 22 1026 1041 10.1183/09031936.03.00089403 14680096

[B11] Maarsingh EJ van Eykern LA de Haan RJ Griffioen RW Hoekstra MO van Aalderen WM Airflow limitation in asthmatic children assessed with a non-invasive EMG technique Respir Physiol Neurobiol 2002 133 89 97 10.1016/S1569-9048(02)00130-1 12385734

[B12] Duiverman ML van Eykern LA Vennik PW Koëter GH Maarsingh EJ Wijkstra PJ Reproducibility and responsiveness of a noninvasive EMG technique of the respiratory muscles in COPD patients and in healthy subjects J Appl Physiol 2004 96 1723 1729 10.1152/japplphysiol.00914.2003 14660508

[B13] Miller MR Hankinson J Brusasco V Burgos F Casaburi R Coates A Crapo R Enright P van der Grinten CP Gustafsson P Jensen R Johnson DC MacIntyre N McKay R Navajas D Pedersen OF Pellegrino R Viegi G Wanger J ATS/ERS Task Force Standardisation of spirometry Eur Respir J 2005 26 319 338 10.1183/09031936.05.00034805 16055882

[B14] Wechsler ME Kelley JM Boyd IO Dutile S Marigowda G Kirsch I Israel E Kaptchuk TJ Active albuterol or placebo, sham acupuncture, or no intervention in asthma N Engl J Med 2011 365 119 126 10.1056/NEJMoa1103319 21751905 PMC3154208

[B15] Tattersfield A McNicol M Salbutamol and isoproterenol – A double-blind trial to compare bronchodilator and cardiovascular activity N Engl J Med 1969 281 1323 1326 10.1056/NEJM196912112812402 4901454

[B16] Dupont WD Plummer WD Power and sample size calculations: a review and computer program; controlled clinical trials Control Clin Trials 1990 11 116 128 10.1016/0197-2456(90)90005-M 2161310

[B17] Driessen J Becker M Westbroek J Van der Wulp A The effect of inspiratory muscle training on sub maximal exercise performance of elite speed skaters Am J Respir Crit Care Med 2012 185 A2055

[B18] Bolger C Tufvesson E Sue-Chu M Devereux G Ayres JG Bjermer L Kippelen P Hyperpnea-induced bronchoconstriction and urinary CC16 levels in athletes Med Sci Sports Exerc 2011 43 1207 1213 10.1249/MSS.0b013e31820750d8 21131866

